# Optimization of culture condition for enhanced decolorization and degradation of azo dye reactive violet 1 with concomitant production of ligninolytic enzymes by *Ganoderma cupreum* AG-1

**DOI:** 10.1007/s13205-012-0079-z

**Published:** 2012-08-07

**Authors:** Mayur Gahlout, Shilpa Gupte, Akshaya Gupte

**Affiliations:** 1 Natubhai V. Patel College of Pure and Applied Sciences, Vallabh Vidyanagar, 388 120 Gujarat India; 2 Ashok and Rita Patel Institute of Integrated Study and Research in Biotechnology and Allied Sciences, New Vallabh Vidyanagar, 388 121 Gujarat India

**Keywords:** *Ganoderma cupreum* AG-1, Reactive violet 1, Laccase, MnP, Decolorization

## Abstract

The strain *Ganoderma cupreum* AG-1 (Genbank accession no. HQ328947) isolated from the decayed wood was evaluated for its ability to decolorize azo dye reactive violet 1 as well as for the production of ligninolytic enzymes. In the initial decolorization study, the strain was capable of decolorizing 19 different azo dyes. The strain was capable of decolorizing dye over a pH range of 4.5–6 at 30 °C. The optimum pH was found to be 4.5. Various other process parameters like additional carbon and nitrogen source and initial dye concentration were also optimized. The decolorization medium was supplemented with appropriate nitrogen source (yeast extract, 5 g l^−1^) and carbon source (mannose, 2 g l^−1^); the decolorization obtained was 98 %. The pattern of enzymes involved in the biodegradation was studied and laccase and MnP were found to be the major enzymes. High laccase activity shown by *G. cupreum* AG-1 and its ability to decolorize dyes are a good indication of its possible use in the treatment of textile effluents.

## Introduction

Environmental pollution has increased with increasing industrial development (Osman et al. 2006). Contamination of water by wide range of pollutants is a serious environmental problem due to their potential human toxicity. The textile finishing generates large amount of dyes, pigments, dispersing agents, salts, leveling agents and heavy metals (Noroozi et al. 2007). The total world colorant production is estimated to be about 8,00,000 tons per year, and at least 10–15 % of the dyes/dyestuffs are released into the environment through the effluents (Revanker and Lele 2007). Owing to their chemical structures, dyes are resistant to fading on exposure to light, water and many chemicals and are difficult to be eliminated by conventional chemical and biological waste treatment methods, due to the effluents high degree of polarity.

Decolorization of dye waste water is a challenging process to the textile industry, and the great potential of microbial decolorizing can be adopted as an effective tool. In the recent past there has been an intensive research on bioremediation of dyes, and the use of white rot fungi/ligninolytic fungi is turning into a promising alternative to replace or supplement present treatment processes (Dos-Santos et al. 2004; Asgher et al. 2006). The abilities of white rot fungi in mineralization of xenobiotics to CO_2_ and water through the highly oxidative and nonspecific ligninolytic system are well documented, which are also responsible for decolorization and degradation of wide range of dyes (Boer et al. 2004; Patel et al. 2009) The extracellular enzyme system includes Laccase (E.C. 1.10.3.2), Manganese peroxidase (MnP, E.C. 1.11.1.13), Lignin peroxidase (LiP, E.C. 1.11.1.14) that are able to decolorize various dyes of different chemical structures (Levin et al. 2004).

The present study aims to enhance the ability of the white rot fungi *Ganoderma cupreum* AG-1 to decolorize various azo dyes. In order to exploit the potential of the new isolates various process parameters like carbon, nitrogen, initial dye concentration, pH and temperature were optimized to develop an economic decolorization process. Furthermore, relationship between ligninolytic enzyme production and decolorization of reactive azo dye by *G. cupreum* AG-1 was assessed. Degradation analysis was studied using UV–Visible, HPTLC and FTIR.

## Materials and methods

### Chemicals and dyes

Ortho-dianisidine was purchased from Sd-fine chemicals (Mumbai, India). 2,2-Azino-bis (3-ethylbenzthiozoline-6-sulphonic acid) (ABTS) was purchased from Sigma, (St. Louis, MO, USA). 2,6-Dimethoxyphenol (DMP) was purchased from Lancaster (Lancs, UK). All other solvents and chemicals used were of analytical grade, unless otherwise stated. The Dyes used in this study were obtained from Meghmani Enterprise Pvt. Ltd., Ahemadabad, Gujarat, India.

### Screening, isolation and identification of fungal strain

Various fruiting bodies from the rotted wood samples were collected from different biotopes in Anand, Gujarat, India. A small portion of wood sample was transferred in plates containing 2 % malt extract agar amended with chloramphenicol (0.5 g l^−1^) and incubated at 30 °C. The cultures isolated were subcultured on modified Sabaroud dextrose agar (SDA) containing (g l^−1^): glucose 20; peptone 10; NaCl 2.5; agar–agar 30; and penicillin G 0.06; streptomycin sulfate, 0.0001. After 7 days of incubation at 30 °C, fungal cultures were transferred on the same medium without antibiotics until pure colonies were obtained. Fungal cultures having similar microscopic characteristics were selected for storage. SDA incorporated with 0.1 g l^−1^ ortho-dianisidine was used for the screening of phenol oxidase. Further confirmation of potential isolates for phenol oxidase activity was carried out using SDA plate containing 0.1 g l^−1^ ABTS and 0.1 g l^−1^ Guaiacol as a chromogenic substrate. The selected isolate was further identified using ITS 1 and ITS 2 gene sequencing. Potential isolate was subculutred and maintained at 4 °C on malt extract agar slants.

### Decolorization studies

Erlenmeyer flasks (250 ml) containing 100 ml of basal culture medium containing (g l^−1^) Glucose 5.0, Yeast Extract 0.5, MgSO_4_·7H_2_O·0.5, KH_2_PO_4_ 0.5, KCl 0.5 were autoclaved at 121 °C for 15 min, cooled and then inoculated with 8 agar plugs of size 8 mm diameter punched from the leading edge of pre-grown fungal culture on MEA plates and incubated at 30 °C for 4 days. On the 5th day of incubation 100 μl of sterilized dye (10,000 ppm in water) was added to the flasks and incubated for 4 days at 30 °C. Uninoculated nutrient medium served as control. Dye decolorization was determined spectrophotometrically by monitoring the absorbance of samples at λ max of the respective dyes using a UV–Visible spectrophotometer (Shimadzu UV 1800, Japan). Results are reported as the mean amount of decolorization for three replicates. The decolorization expressed in % of the initial dye concentration was calculated as follows:where *A*_0_ is the absorbance value of the initial dye concentration and *A*_*t*_ is the absorbance value of the dye concentration in sample at time *t*.

### Sample extraction

A sample of 2 ml from inoculated flasks was withdrawn at a regular interval of 24 h and centrifuged at 10,000 rpm at 4 °C for 15–20 min. The clear supernatant obtained was used as the sample for dye decolorization and Lignin modifying enzyme (LME) activity analysis.

### Enzyme assay

Laccase, MnP and MnIP activity were assayed spectrophotometrically (Shimadzu UV 1800, Japan) in extracted sample. Laccase activity (E.C. 1.10.3.2) was determined by measuring the oxidation of 2,2-Azino-Bis-3-ethyl-benzthiozoline-6-sulphonic acid (ABTS) at 420 nm as described by Niku et al. (1990). Manganese peroxidase (MnP, E.C. 1.11.1.13) activity was measured by oxidation of 2,6-dimethoxy phenol (DMP) at 469 nm and MnP activity was corrected for manganese-independent peroxidase (MnIP) activity by subtracting the MnIP activity obtained at pH 3.25 in absence of MnSO_4_ at 469 nm as described by Martinez et al. (1996). One unit of enzyme activity was defined as the amount of enzyme that oxidized 1 μM of substrate per min at room temperature.

### Optimization of process parameters for enhanced decolorization of reactive violet 1 (RV 1) dye

A traditional stepwise strategy was adopted for the optimization of the decolorization of RV 1 dye by varying one factor at a time. All the decolorization experiments were performed in triplicates.

### Effect of static and shaking condition

The flask containing 100 ml medium was inoculated with 8 agar plugs supplemented with 100 ppm RV 1 dye and incubated at static and shaking condition (150 rpm) at 30 °C for 4 days. Abiotic control was also kept and the % decolorization was measured as mentioned earlier.

### Effect of carbon and nitrogen source

During this study the organism was supplemented with additional carbon and nitrogen sources. To enhance decolorization performance of RV 1, the decolorization medium was supplemented with different carbon sources like glucose, fructose, mannose, xylose, starch, lactose and mannitol, individually added at a concentration of 5 g l^−1^. Similarly, in another set various organic nitrogen sources (yeast extract, peptone, urea) and inorganic nitrogen sources (Ammonium sulfate, ammonium nitrate, ammonium ferric citrate) were added at a concentration of 5 g l^−1^ to the medium to study the effect on decolorization process.

### Effect of physicochemical parameters

Physicochemical parameters like temperature (25–45 °C) and pH (3–10) were monitored to study the effect on decolorization of RV 1 dye. The flask containing 100 ml medium was inoculated with 8 agar plugs supplemented with 100 ppm dyes and incubated at 30 °C under shaking conditions. Abiotic control (without culture) was also kept and the decolorization efficiency was measured as mentioned earlier.

### Effect of initial dye (RV 1) concentrations

In order to examine the effect of initial dye concentration on decolorization under shaking condition, 0.1–5 g l^−1^ of RV 1 was added to the medium and inoculated with 8 agar plugs and incubated at 30 °C. The % decolorization and ligninolytic enzyme production was determined after 4th day of incubation. Abiotic control (without culture) was always included.

### Decolorization of repeated addition of (RV 1) dye aliquots

Repetitive decolorizing ability of the culture *G. cupreum* AG-1 was studied by repeated spiking of dye (100 mg l^−1^) at each cycle. After each decolorization cycle, samples were withdrawn for percent decolorization with respect to time of each cycle.

### Degradation analysis

Decolorization was monitored by UV–Visible spectroscopic analysis (Shimadzu UV 1800, Japan), whereas biodegradation was monitored using HPTLC and FTIR spectroscopy. The HPTLC analysis was carried out using precoated silica gel 60 F254 plate (Merck, Germany). A 5 μl of the sample was spotted on TLC plates using micro syringe (HPTLC, Camag, Linomat 5). The solvent system used was n-propanol:ethyl acetate:methanol:water: 2:1:1:1. The dye chromatogram was observed by exposing to the ultraviolet light (254 nm) and in visible light using Camag TLC scanner 3. The biodegradation of RV 1 was further characterized by FTIR spectroscopy (Fourier transform infrared spectroscopy, Perkin Elmer Spectra GX). The FTIR analysis was carried out in the mid IR region of 400–4,000 cm^−1^. The control and degraded dye samples were mixed with spectroscopically pure KBr in the ratio of 5:95 to form a uniform pellets, which was then fixed in sample holder, and the analysis was carried out.

## Results and discussion

### Isolation and identification of ligninolytic fungi

**Fig. 1:**
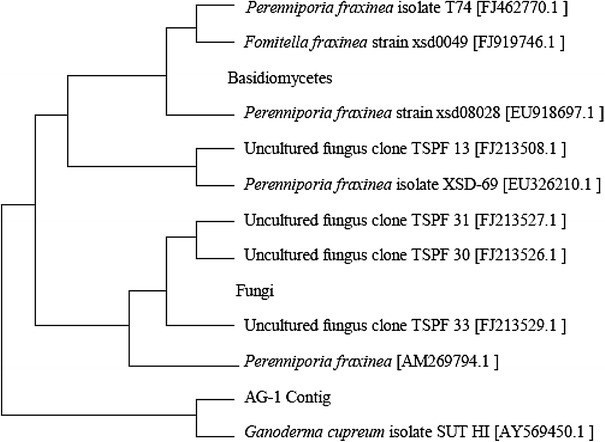
Phylogenetic dendogram for *G. cupreum* AG-1 based on ITS 1 and ITS 2 gene sequencing

A total of 25 fruiting bodies were collected and screened for the detection of LME using Ortho-dianisidine (0.1 g l^−1^) as a chromogen on SDA plates. The dark brown colored zone surrounding the agar plug of fungi on SDA Ortho-dianisidine plate is an indication of Bevandam’s reaction. Nine isolates showing positive Bevandam reaction were further confirmed for the presence of phenol oxidase on ABTS (0.1 g l^−1^) and Guaiacol (0.1 g l^−1^) supplemented SDA plates. The culture designated as AG-1, from the positive nine isolate, was found to be substantial producer of phenol oxidase as compared with other eight isolates. The mycelium of AG-1 shows green color zone on ABTS (0.1 g l^−1^) SDA plates and brown color zone on guaiacol (0.1 g l^−1^) SDA plates. The phenol oxidase producing strain AG-1 was thus selected for our further study. Morphological examination of AG-1 shows the strain grew well, covering the entire petri-plate surface in 8–10 days. The isolate was found to be non-sporulating, with abundant clamp connection forming in mycelia, which are characteristics of basidiomycetes fungi. The identification of AG-1 culture was further corroborated by studies on its ITS 1 and ITS 2 gene sequences carried out by Xplorigen, Pvt. Ltd. (New Delhi, India). Blast similarity search analysis based on ITS 1 and ITS 2 gene sequences revealed that isolates belong to the genus *Ganoderma*. The closest Phylogenetic neighbor was found to be *G. cupreum* SUT HI (AY 569450.1) with 86 % homology. Phylogenetic relationship could be inferred through the alignment and cladistic analysis of homologues nucleotide sequences of known fungi (Fig. 1). The gene sequences have been deposited in the Gen Bank database of NCBI under accession number EU 867248 (1,147 bp). The strain *Ganoderma* sp. AG-1 was thus identified as *G. cupreum* AG-1.

### Dye decolorization experiments in liquid medium

The *G. cupreum* AG-1 was tested for its ability to decolorize various textile dyes (Table 1) as waste water from the textile industries containing mixture of dyes. It was found that the strain was able to decolorize variety of different dyes within 4–8 days, with as low as 18 % (reactive red BS) to a maximum of 91 % (RV 1) decolorization. The variation in decolorization efficiency and the time required for decolorization may be due to molecular complexity of the dyes, culture conditions and the enzyme system produced by the fungi (Katia et al. 2006; Santos et al. 2006). A slower rate of decolorization can also be attributed to higher molecular weight and the presence of inhibitory group like –NO_2_ and –SO_3_ groups in the dyes (Hu and Wu 2001). Xiu et al. (2009) reported Decolorization of azo, triphenylmethane and anthraquinone dyes, Varied in between 30 and 97 % in 7 days by *Trametes* sp. SQ01 with concomitant production of laccase. Ismat et al. (2010) reported Novosal direct dyes’ decolorization by *Agaricus biotrocus* A66 which varied in the region of 26–78 % in 4 days. Katia et al. (2006) reported decolorization of 28 reactive dyes using *Trametes villosa* and *P. sanguineus*.

**Table 1:** Decolorization of textile dyes by *G. cupreum* AG-1 in liquid medium

Sr. No	Dyes	λ_max_	Decolorization %	Time of incubation (days)
1.	Acid green	617	86	6
2.	Green ME4BL	639	31	5
3.	Reactive red M5B	534	71	8
4.	Reactive red ME4BL	534	45	5
5.	**Reactive violet-1**	**517**	**91**	4
6.	Reactive G yellow MR	414	32	8
7.	Reactive red BS	517	18	8
8.	Reactive yellow GR	413	82	8
9.	Reactive blue 3R	577	74	6
10.	Yellow 3RW	419	21	7
11.	Red-10	567	26	4
12.	Acid red-131	531	62	5
13.	Di-red	521	56	5
14.	Reactive red HE8D	548	43	8
15.	Reactive black 31	568	67	6
16.	Reactive black V5B	601	45	6
17.	Reactive violet 5	560	23	5
18.	Reactive blue M4GD	616	57	4
19.	Acid blue	617	71	8

Bold value indicates the maximum decolorized dye Reactive Violet 1, thus selected as model azo dye

### Effect of static and shaking condition on decolorization process

In order to evaluate the decolorization efficiency of *G. cupreum* AG-1 the flasks were incubated under shaking condition (150 rpm) and static condition at 30 °C. Shaking/agitated condition favored the decolorization of RV 1 with about 90 % decolorization being obtained in 4 days, whereas, 73 % decolorization was obtained under static condition in 4 days, thus suggesting shaking condition is favorable for decolorization. The higher % decolorization obtained under shaking condition may be attributed to increase in biomass and oxygen transfer between the cells and medium. In the present study no residual color was observed on the biomass, suggesting that the decolorization and degradation may have occurred because of the action of the ligninolytic enzymes produced by the culture during the decolorization process. To confirm whether the decolorization occurred due to change in pH or not, change in pH was recorded which was in the range of 4.5–5.5 under shaking as well as static condition. The pH was almost similar to the pH of the original medium thus suggesting the action of the ligninolytic enzymes. Jarsoz et al. (2002) reported agitated cultures of *Bjerkandera fumosa*, *Kuehneromycesmutabilis and Strofaria rugoso*-*annulata* for 100 % decolorization of Acid red 183 dye and 75–100 % decolorization of Basic Blue 22 and Basic Blue 20 dyes, respectively.

### Effect of carbon and nitrogen source on decolorization process

**Fig. 2:**
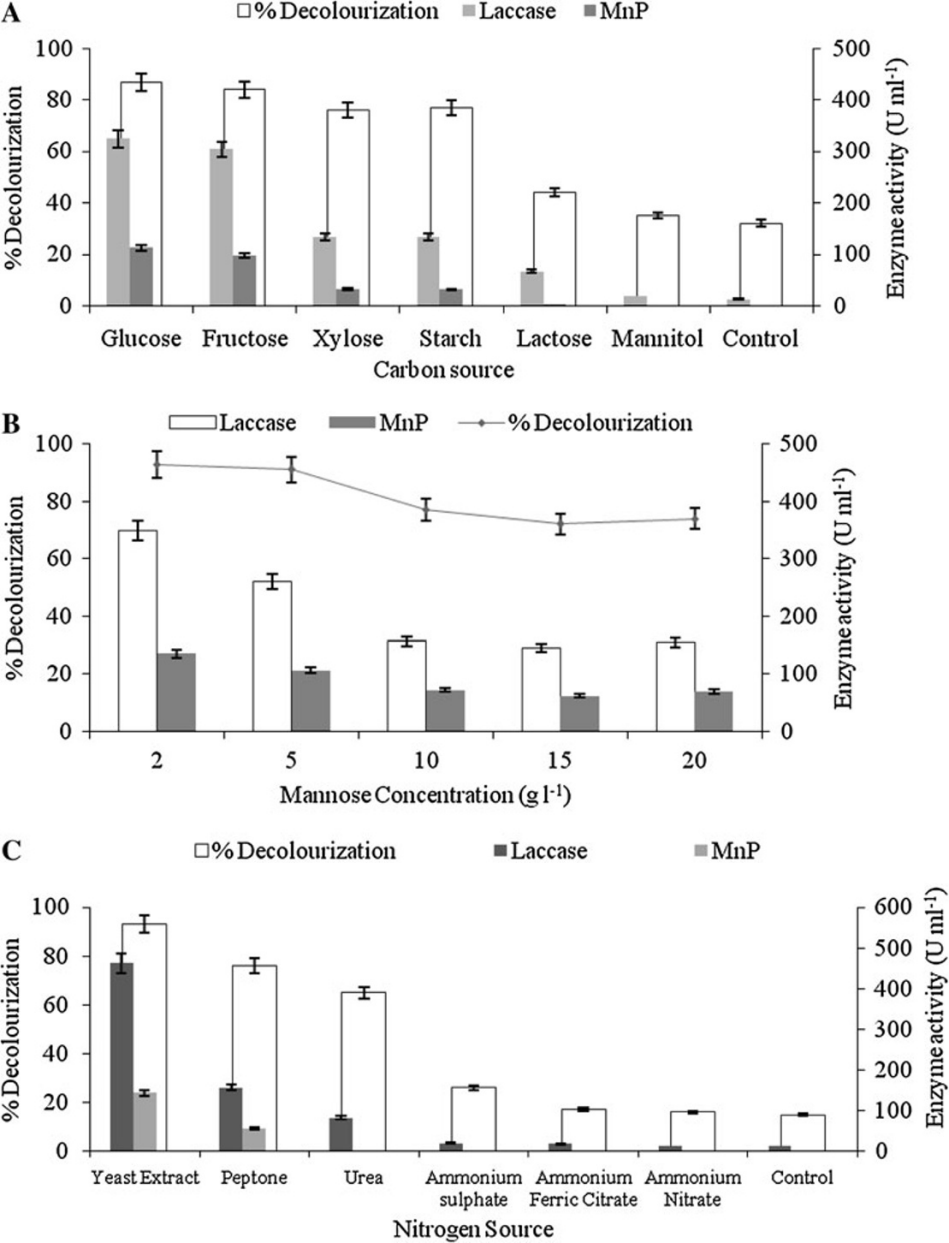
Effect of **a** carbon source, **b** mannose concentration and **c** nitrogen source on decolorization of RV 1 dye by *G. cupreum* AG-1

To increase the decolorization efficiency the decolorization medium was supplemented with different carbon sources (glucose, fructose, mannose, xylose, starch, lactose and mannitol) at a concentration of 5 g l^−1^ (Fig. 2a). Maximum decolorization obtained was 91 % in the flask receiving mannose as an additional carbon source; following mannose, medium supplemented with glucose and fructose showed higher dye decolorization efficiency as compared with other carbon sources used. Laccase was the major enzyme (371 U ml^−1^) produced in the flask receiving mannose as an additional carbon source as compared with MnP (118 U ml^−1^). Ligninolytic enzyme production of *G. cupreum* AG-1 with various carbon sources was also consistent with the decolorization pattern. The decolorization ability of *G. cupreum* AG-1 decreased in the absence of the carbon source. This suggests that the dye molecule does not serve as a sole source of carbon and an addition of external co-substrate seems to be effective to promote the decolorization of RV 1 dye (Fig. 2a). Fazli et al. (2010) reported glycerol as best carbon source for maximum decolorization (95.3 %) of reactive blue dye by *Ganoderma* species on the 5th day of incubation. Furthermore, the effect of different mannose concentrations (2–20 g l^−1^) on dye decolorization was also studied; maximum dye decolorization 91–93 % was obtained with a concentration of 2 g l^−1^. Figure 2b depicts, with an increase in concentration of mannose, a decrease in dye decolorization, and ligninolytic enzyme production was observed. This may be because higher concentration of mannose may create highly acidic condition or catabolite repression leading to a negative effect on dye decolorization. The results obtained are in accordance with other reports wherein carbon-limited conditions trigger ligninolytic enzyme activity in white rot fungi, which is required for pollutant degradation (Kapdan and Kargi 2002).

In case of nitrogen source Fig. 2c depicts a maximum of 96 % decolorization with laccase and MnP activity of 462 and 144 U ml^−1^, respectively, on the 4th day of incubation when yeast extract was used as nitrogen sources. Peptone and urea showed lower efficiency for dye decolorization than yeast extract. However, the uses of inorganic nitrogen sources were found to be less efficient in the decolorization of RV 1 dye. The result obtained is in agreement with those of Saratale et al. (2009) who studied addition of yeast extract in synthetic media that showed maximum decolorization of Navy blue HER by *Trichosporon beigelii*. Ponraj et al. (2011) also found yeast extract as the most effective nitrogen source for the decolorization of True blue dye by *Aspergillus flavus*. However, Kashif et al. (2011) reported ammonium sulfate as the best nitrogen source for maximum decolorization (89.1 %) of Sollar Golden Yellow R dye with 253.67 U ml^−1^ of laccase activity by *Pleurotus osttreatus* species. The presence of various carbon and nitrogen sources in the medium might be leading to stimulatory or inhibitory effect on the induction of enzyme system, involved in decolorization of RV 1 dye resulting in the variation of % decolorization.

### Effect of pH on decolorization process

**Fig. 3:**
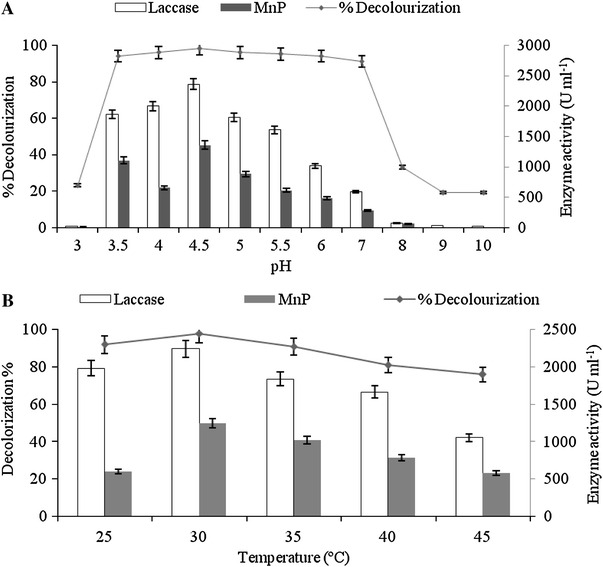
Effect of **a** pH and **b** temperature on decolorization of RV 1 dye by *G. cupreum* AG-1

The pH of the culture medium is critical to the growth, metabolic activity, ligninolytic enzyme production and xenobiotic pollutant degradation by white rot fungi. To investigate the effect of pH on RV 1dye decolorization by *G. cupreum* AG-1, the initial pH of the medium was adjusted in the range of 3–10. The results obtained indicate that *G. cupreum* AG-1 was capable of decolorizing RV 1 dye efficiently between pH range of 4.5–6. Maximum decolorization (98 %) was obtained at pH 4.5 with high activity of Laccase (2,358 U ml^−1^) and MnP (1,352 U ml^−1^) enzyme activity (Fig. 3a), thus suggesting that acidic condition favors RV 1 dye decolorization. However, negligible decolorization was observed at pH 3 and pH 7–10, as varying low and high pH lead to inhibitory effect on the growth of the microorganisms. At high pH values, reactive dye solutions are more negatively charged, and dye removal efficiency by white rot fungi is readily decreased (Tak et al. 2004). Various researchers reported optimum ligninolytic enzyme production as well as dye decolorization capabilities in acidic pH range between 4 and 6. (Mazmanci and Ali 2010; Shazia and Safia 2011; Ali and Mohamedy 2012).

### Effect of temperature on decolorization process

Incubation temperature is an important process parameter that varies from one microorganism to other microorganisms. A slight change in the incubation temperature may affect the growth and enzyme activities of microorganisms. The effect of varying incubation temperatures on decolorization of RV 1 by *G. cupreum* AG-1 is shown in Fig. 3b. The fungus exhibited better dye decolorization in the lower incubation temperature ranges (25–35 °C). The maximum decolorization (98 %) was observed in the shake flask incubated at 30 °C for 4 days with 2,244 U ml^−1^ laccase and 1,244 U ml^−1^ MnP. The dye decolorization ability of fungal culture was found to decrease with an increase in incubation temperature up to 45 °C. Similar results were also shown by Abedin (2008) for the decolorization of crystal violet and malachite green by *Fusarium solani.* Haq et al. (2008) also reported 30 °C as an optimum temperature for the decolorization of Cibacron Red FN-2BL dye by *Schizophyllum commune* IBL 6.

### Effect of varying dye (RV 1) concentrations

**Fig. 4:**
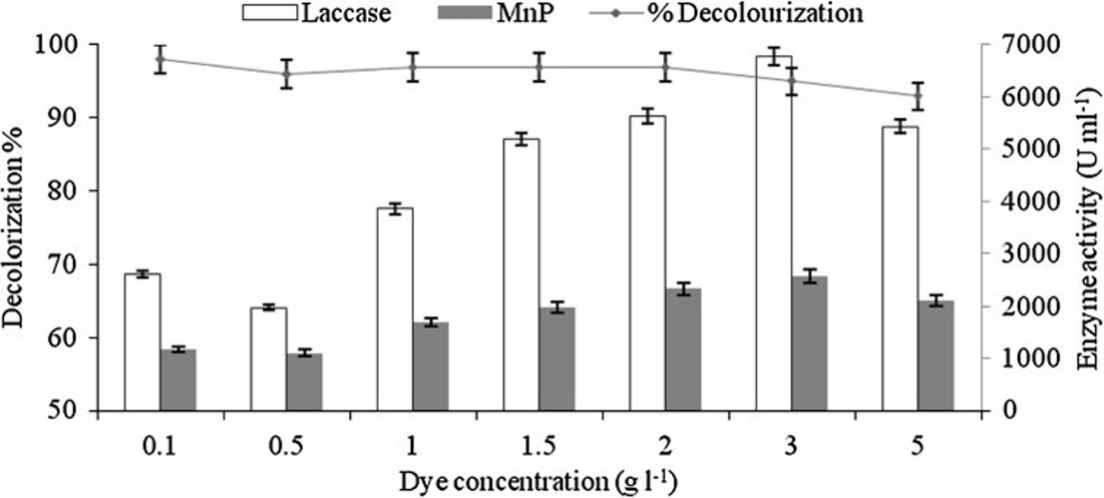
Effect of varying dye concentrations decolorization of RV 1 dye by *G. cupreum* AG-1

Actual concentration of the dyes in dye house effluent have been reported to a range from 60 to 250 mg l^−1^ (Bhatt et al. 2005) In the present study different concentrations of the dye were used to investigate the effect of initial dye concentrations on activities of laccase, MnP and dye removal efficiency under optimum conditions. The dye solutions were varied with dye concentration increasing from 0.1 to 5.0 g l^−1^. The dye was well tolerated and decolorized by the fungus in all the tested dye concentrations. It was observed that at lower concentrations, the % decolorization was higher and reached up to 98 % with the lowest concentration of 0.1 g l^−1^. When concentration of dye was increased from 0.1 to 5.0 g l^−1^, the decolorization efficiency of organism decreased marginally from 98 to 93 % (Fig. 4). However, the ligninolytic enzymes activity increased within increase in dye concentration up to 3 g l^−1^ (Laccase and MNP activity was 2,607 and 1,167 U ml^−1^ at 0.1 g l^−1^ dye concentration whereas at 3 g l^−1^, Laccase and MNP activity reached to 6,788 and 2,583 U ml^−1^, respectively). This suggests that the higher concentration of dye induces the ligninolytic enzyme production which in turn results in the decolorization of RV 1 dye. Vaithanomast et al. (2010) reported the induction of different types of ligninolytic enzyme depends on the structure of dye. Laccase activity in all the system flasks was much higher than MnP, which indicates that laccase could be mainly responsible for the dye decolorization process. Kashif et al. (2011) reported 78 % decolorization of 0.5 g l^−1^ of solar golden yellow R dye by *Pleurotous Ostreatus*. Vaithanomast et al. (2010) reported more than 90 % decolorization of 1.0 g l^−1^ reactive dye RBBR and RB5 by *Datronia* species, whereas Shazia and Safia (2011) denoted 0.2 g l^−1^ of AR151 dye as maximum limit to be decolorized (60–70 %) by three indigenous *Aspergillus* species.

### Effect of repeated addition of dye

**Fig. 5:**
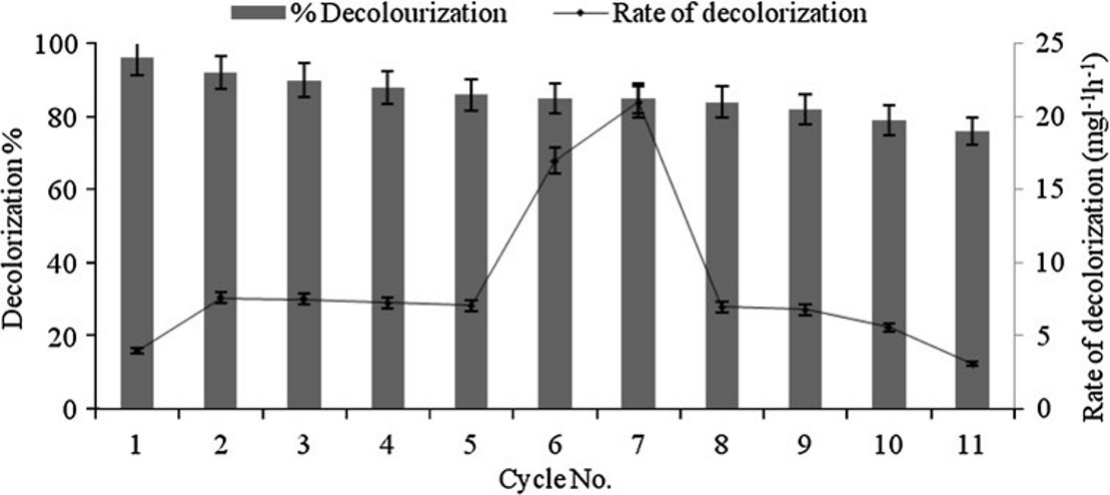
Effect of repeated addition of dye aliquots on decolorization of RV 1 dye by *G. cupreum* AG-1

Repeated dye decolorization of RV 1 was studied under optimized condition. The prime intention of this study was to evaluate the efficiency of our fungal isolate to decolorize RV 1 dye by repeated addition of 100 mg l^−1^ RV 1 dye. Fig. 5 shows that *G. cupreum* AG-1 was able to decolorize RV 1 dye consecutively up to 11 cycles with gradual reduction in decolorization efficiency ranging from 96 to 70 % for 1–11 cycles, respectively. In first cycle, 96 % decolorization was obtained within 24 h. The subsequent addition of dye results in faster rate of decolorization process till the 7th cycle. Maximum decolorization rate (21 mg l^−1^ h^−1^) was obtained at the 7th cycle of decolorization process. From 8th cycle to last cycle the decolorization efficiency of organism gradually decreased and required more time for decolorization of RV 1 dye. The eventual cessation of decolorization may be obtained due to the nutrient depletion in system flasks that may lead to the unstable enzyme production and uncontrolled growth of fungal mycelium (Saratale et al. 2006). Jadhav et al. (2009) showed six cycles of decolorization process by *Comamonas* sp UVS with more than 70 % decolorization of direct blue GLL dye,whereas Saratale et al. (2009) reported four cycles of Navy blue HER dye decolorization by *Trichosporon beigelii*.

### Degradation analysis

**Fig. 6:**
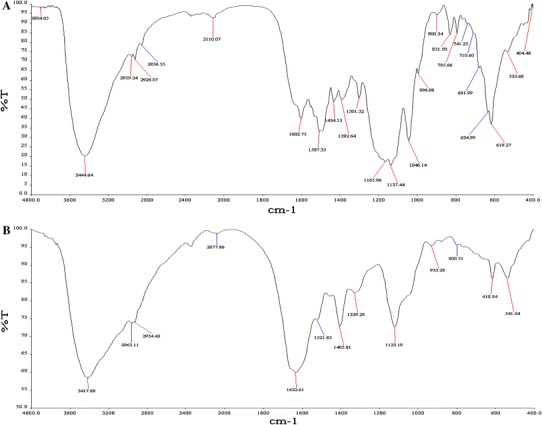
FTIR analysis of RV 1 dye (**a**) and its degraded product (**b**) by *G. cupreum* AG-1

UV–Visible analysis has been used to confirm the decolorization of dyes. In the present study decolorization of RV 1 was due to the biodegradation and is not a merely a visible decolorization. Spectrophotometric analysis of RV 1 showed a maximum absorbance at 560 nm and decrease in absorbance of samples withdrawn after decolorization using *G. cupreum* AG-1. If the removal of dye is attributed to the process of biodegradation, a major visible light absorbance peak would completely disappear or a major peak will appear Chen et al. (2003). The result obtained indicates that color removal may be largely due to biodegradation. Similar results were also reported by Saratale et al. (2009). Furthermore, the appearance of four different spots in the degraded sample with *R*_*f*_ values of 0.90, 0.85, 0.73, 0.27 as compared with the *R*_*f*_ value (0.93) of original dye confirming the degradation of RV 1 dyes. The FTIR spectrum of control dye compared with degraded product is as shown in Fig. 6a and b. In case of control dye (Fig. 6a) peak around 1,500 cm^−1^, assigned to (N=N) Azo-bond vibration, peaks around 1,602 and 1,434 cm^−1^ may be characteristics of C=C aromatic skeletal vibration and azo linkages (–N=N–) on aromatic structures, bands at region 2,960–2,850 cm^−1^ originate from CH_3_ asymmetric, CH_3_ symmetric vibrations and CH_2_ asymmetric stretching vibration. The peaks at region 1,160–1,040 cm^−1^ can be assigned to CO, CN or phenolic C–O vibration. The FTIR spectrum of degraded product (Fig. 6b) showed major changes in the finger print region. The FTIR spectra of degraded metabolite by *G. cupreum* AG-1 display a peak at 1,632 cm^−1^ which points towards the formation of C=O stretching. This C=O group may be generated by the oxidizing action of laccase enzyme which may have converted the aromatic structure to quinone-like structure. The peak generated at 1,405, 1,521, 1,123 cm^−1^ represents O=H stretching, nitroalkane and C–O–C stretching, The FTIR pattern of degraded metabolite does not have a peak at 1,500 cm^−1^, which indicates the loss Azo bond. Generation of similar peaks has also been reported for different dyes by other researchers (Kalme et al. 2007; Rajeshwari et al. 2011; Jadhav et al. 2007; Kumar et al. 2010; Beata et al. 2003).

The isolate *G. cupreum* AG-1 has an excellent potential for bioremediation of reactive violet-1 dye. The culture can efficiently decolorize the dye at a high concentration up to 5 g l^−1^ under acidic pH, using mannose as additional carbon source and yeast extract as nitrogen source. Although high concentration of dyes might have toxic effect on fungi, it was found that even at a high concentration of 5 g l^−1^, the reactive dye was tolerated and degraded by the *G. cupreum* AG-1. Laccase seems to play a major role for oxidative breakdown of RV 1 dye. Hence the indigenous strain could be utilized for the treatment of dye containing waste water.
